# Supplementary material for: Multi‐dataset identification of innovative feature genes and molecular mechanisms in keratoconus
Source: J Cell Mol Med. 2024 Sep 19;28(18):e70079. doi: 10.1111/jcmm.70079 (PMC11412914; doi:10.1111/jcmm.70079)
Supplement: Supplementary file 2 — Material S2. [file JCMM-28-e70079-s002.zip › 7.GSEA analysis/4.MYRF/KEGG_MELANOGENESIS.html]

Details for gene set KEGG\_MELANOGENESIS[GSEA]

|  || Dataset | MYRF.group.cls#h\_versus\_l |
| Phenotype | group.cls#h\_versus\_l |
| Upregulated in class | l |
| GeneSet | KEGG\_MELANOGENESIS |
| Enrichment Score (ES) | -0.41062486 |
| Normalized Enrichment Score (NES) | -1.5572649 |
| Nominal p-value | 0.01048218 |
| FDR q-value | 0.20091265 |
| FWER p-Value | 0.674 |
Table: GSEA Results Summary

  

Fig 1: Enrichment plot: KEGG\_MELANOGENESIS      
 Profile of the Running ES Score & Positions of GeneSet Members on the Rank Ordered List

  

| SYMBOL | TITLE | RANK IN GENE LIST | RANK METRIC SCORE | RUNNING ES | CORE ENRICHMENT || 1 | CAMK2B | na | 283 | 0.659 | 0.0317 | No |
| 2 | ADCY1 | na | 1675 | 0.385 | 0.0149 | No |
| 3 | ADCY2 | na | 2688 | 0.312 | 0.0046 | No |
| 4 | MC1R | na | 3035 | 0.293 | 0.0123 | No |
| 5 | ASIP | na | 3226 | 0.284 | 0.0240 | No |
| 6 | CAMK2A | na | 4292 | 0.245 | 0.0081 | No |
| 7 | ADCY5 | na | 4506 | 0.238 | 0.0163 | No |
| 8 | CALML6 | na | 5567 | 0.206 | -0.0018 | No |
| 9 | WNT10B | na | 5576 | 0.206 | 0.0104 | No |
| 10 | CALML5 | na | 7292 | 0.156 | -0.0297 | No |
| 11 | WNT16 | na | 8449 | 0.128 | -0.0553 | No |
| 12 | ADCY7 | na | 8605 | 0.125 | -0.0522 | No |
| 13 | FZD1 | na | 9008 | 0.116 | -0.0568 | No |
| 14 | WNT5A | na | 9048 | 0.115 | -0.0510 | No |
| 15 | KRAS | na | 9371 | 0.108 | -0.0537 | No |
| 16 | CREB3L3 | na | 9452 | 0.107 | -0.0496 | No |
| 17 | PLCB3 | na | 9511 | 0.106 | -0.0448 | No |
| 18 | CREBBP | na | 9688 | 0.102 | -0.0438 | No |
| 19 | PLCB2 | na | 9762 | 0.100 | -0.0398 | No |
| 20 | ADCY4 | na | 9919 | 0.097 | -0.0384 | No |
| 21 | GNAI3 | na | 9952 | 0.097 | -0.0335 | No |
| 22 | FZD3 | na | 10112 | 0.094 | -0.0324 | No |
| 23 | CREB3L1 | na | 10715 | 0.083 | -0.0447 | No |
| 24 | CAMK2G | na | 10986 | 0.078 | -0.0478 | No |
| 25 | PRKX | na | 11663 | 0.068 | -0.0632 | No |
| 26 | FZD5 | na | 11924 | 0.065 | -0.0668 | No |
| 27 | CALM2 | na | 11947 | 0.064 | -0.0635 | No |
| 28 | WNT3 | na | 12453 | 0.057 | -0.0747 | No |
| 29 | FZD4 | na | 13390 | 0.045 | -0.0990 | No |
| 30 | ADCY6 | na | 13405 | 0.045 | -0.0966 | No |
| 31 | GNAQ | na | 13411 | 0.045 | -0.0941 | No |
| 32 | WNT2 | na | 13551 | 0.043 | -0.0955 | No |
| 33 | MAP2K2 | na | 14012 | 0.038 | -0.1065 | No |
| 34 | PRKCA | na | 14202 | 0.036 | -0.1098 | No |
| 35 | MAPK3 | na | 14382 | 0.034 | -0.1129 | No |
| 36 | TCF7L2 | na | 15138 | 0.027 | -0.1331 | No |
| 37 | GNAI2 | na | 15256 | 0.025 | -0.1349 | No |
| 38 | FZD9 | na | 15491 | 0.023 | -0.1403 | No |
| 39 | CTNNB1 | na | 15600 | 0.022 | -0.1421 | No |
| 40 | CREB3 | na | 15923 | 0.018 | -0.1503 | No |
| 41 | PRKCG | na | 15973 | 0.018 | -0.1506 | No |
| 42 | CALML3 | na | 17129 | 0.008 | -0.1835 | No |
| 43 | PLCB4 | na | 17554 | 0.004 | -0.1955 | No |
| 44 | ADCY9 | na | 17667 | 0.003 | -0.1986 | No |
| 45 | CALM1 | na | 17693 | 0.002 | -0.1992 | No |
| 46 | WNT8A | na | 17911 | 0.000 | -0.2054 | No |
| 47 | CREB3L2 | na | 18517 | -0.005 | -0.2226 | No |
| 48 | GNAO1 | na | 18564 | -0.006 | -0.2235 | No |
| 49 | GSK3B | na | 19466 | -0.016 | -0.2486 | No |
| 50 | FZD6 | na | 19569 | -0.017 | -0.2505 | No |
| 51 | PRKACA | na | 19584 | -0.017 | -0.2499 | No |
| 52 | FZD8 | na | 20046 | -0.022 | -0.2619 | No |
| 53 | ADCY8 | na | 20359 | -0.026 | -0.2693 | No |
| 54 | MAPK1 | na | 20406 | -0.027 | -0.2690 | No |
| 55 | WNT4 | na | 20821 | -0.031 | -0.2791 | No |
| 56 | WNT3A | na | 20924 | -0.033 | -0.2801 | No |
| 57 | EDNRB | na | 20991 | -0.033 | -0.2799 | No |
| 58 | WNT9A | na | 21202 | -0.036 | -0.2838 | No |
| 59 | CREB3L4 | na | 21651 | -0.041 | -0.2943 | No |
| 60 | KIT | na | 21671 | -0.042 | -0.2923 | No |
| 61 | RAF1 | na | 21720 | -0.042 | -0.2911 | No |
| 62 | HRAS | na | 21856 | -0.044 | -0.2923 | No |
| 63 | DVL1 | na | 22373 | -0.050 | -0.3042 | No |
| 64 | GNAS | na | 22466 | -0.052 | -0.3037 | No |
| 65 | CREB1 | na | 23204 | -0.063 | -0.3212 | No |
| 66 | DVL3 | na | 23247 | -0.063 | -0.3186 | No |
| 67 | KITLG | na | 23576 | -0.069 | -0.3239 | No |
| 68 | CALM3 | na | 23602 | -0.069 | -0.3204 | No |
| 69 | PRKCB | na | 25770 | -0.108 | -0.3764 | No |
| 70 | ADCY3 | na | 26955 | -0.134 | -0.4025 | Yes |
| 71 | WNT1 | na | 27000 | -0.135 | -0.3956 | Yes |
| 72 | EP300 | na | 27396 | -0.145 | -0.3982 | Yes |
| 73 | TCF7L1 | na | 27666 | -0.152 | -0.3968 | Yes |
| 74 | WNT10A | na | 28144 | -0.164 | -0.4006 | Yes |
| 75 | DVL2 | na | 28187 | -0.166 | -0.3918 | Yes |
| 76 | GNAI1 | na | 28414 | -0.173 | -0.3878 | Yes |
| 77 | WNT2B | na | 28930 | -0.189 | -0.3913 | Yes |
| 78 | WNT7B | na | 29404 | -0.203 | -0.3926 | Yes |
| 79 | NRAS | na | 30019 | -0.224 | -0.3968 | Yes |
| 80 | TCF7 | na | 30107 | -0.227 | -0.3856 | Yes |
| 81 | MAP2K1 | na | 30150 | -0.229 | -0.3729 | Yes |
| 82 | FZD10 | na | 30271 | -0.233 | -0.3623 | Yes |
| 83 | POMC | na | 30351 | -0.236 | -0.3503 | Yes |
| 84 | WNT8B | na | 30486 | -0.242 | -0.3395 | Yes |
| 85 | PLCB1 | na | 30796 | -0.254 | -0.3330 | Yes |
| 86 | PRKACG | na | 31385 | -0.285 | -0.3327 | Yes |
| 87 | WNT9B | na | 31651 | -0.298 | -0.3223 | Yes |
| 88 | CAMK2D | na | 31910 | -0.312 | -0.3109 | Yes |
| 89 | WNT5B | na | 32169 | -0.328 | -0.2985 | Yes |
| 90 | WNT7A | na | 32275 | -0.335 | -0.2813 | Yes |
| 91 | FZD2 | na | 32361 | -0.340 | -0.2631 | Yes |
| 92 | DCT | na | 32507 | -0.351 | -0.2461 | Yes |
| 93 | EDN1 | na | 33100 | -0.397 | -0.2391 | Yes |
| 94 | WNT6 | na | 33111 | -0.398 | -0.2153 | Yes |
| 95 | LEF1 | na | 33408 | -0.432 | -0.1977 | Yes |
| 96 | PRKACB | na | 33438 | -0.435 | -0.1722 | Yes |
| 97 | FZD7 | na | 34203 | -0.571 | -0.1596 | Yes |
| 98 | MITF | na | 34465 | -0.663 | -0.1270 | Yes |
| 99 | WNT11 | na | 34512 | -0.683 | -0.0870 | Yes |
| 100 | TYRP1 | na | 34607 | -0.755 | -0.0440 | Yes |
| 101 | TYR | na | 34631 | -0.783 | 0.0028 | Yes |
Table: GSEA details [plain text format]

  

Fig 2: KEGG\_MELANOGENESIS      
 Blue-Pink O' Gram in the Space of the Analyzed GeneSet

  

Fig 3: KEGG\_MELANOGENESIS: Random ES distribution      
 Gene set null distribution of ES for **KEGG\_MELANOGENESIS**
